# Mitochondria-Associated Gene SLC25A32 as a Novel Prognostic and Immunotherapy Biomarker: From Pan-Cancer Multiomics Analysis to Breast Cancer Validation

**DOI:** 10.1155/2024/1373659

**Published:** 2024-04-29

**Authors:** Shiqi Zuo, Siyuan He, Zhiqin Zhu, Yingying Zhang, Yanjie Hou, Ziqing Wu, Yao Tang

**Affiliations:** ^1^Department of Pathology, Southern Medical University Hospital of Integrated Traditional Chinese and Western Medicine, Southern Medical University, Guangzhou, Guangdong 510315, China; ^2^Guangdong Provincial Key Laboratory of Molecular Tumor Pathology, Southern Medical University, Guangzhou 510515, Guangdong, China; ^3^Department of Pathology, School of Basic Medical Science, Southern Medical University, Guangzhou 510515, China; ^4^Department of Hepatology, Southern Medical University Hospital of Integrated Traditional Chinese and Western Medicine, Southern Medical University, Guangzhou, Guangdong 510315, China

## Abstract

**Background:**

Mutations in SLC25A32 in humans cause late-onset exercise intolerance, which is associated with various neurological and metabolic diseases. However, its specific mechanism of action in tumour development is poorly understood owing to the lack of multiomics integrated analysis of SLC25A32 in pan-cancer.

**Methods:**

We used various analytical tools to comprehensively investigate the transcription, protein level, and promoter methylation of SLC25A32. Furthermore, the GSCA and cBioPortal databases were used to evaluate the inheritance impact and epigenetic alterations of SLC25A32 in pan-cancer. SLC25A32 expression and the prognostic significance of copy number alterations in multiple cancers were compared using the UCSCXenaShiny and GEPIA2.0 platforms, and its specific function in breast cancer was experimentally verified.

**Results:**

SLC25A32 is abnormally expressed at the transcriptional and protein levels in most cancer types, with aberrant DNA promoter methylation and significant gene amplification in most tumours. SLC25A32 is significantly associated with the survival prognosis of some cancers, immune infiltrating cells, tumour stemness, and immune-related markers. SLC25A32 knockdown decreased breast tumour cell proliferation, invasion, and metastasis.

**Conclusions:**

This study aimed to reveal SLC25A32 as a novel prognostic biomarker for pan-cancer prediction and immunotherapy efficacy and specifically describes its underlying mechanism of action in breast cancer. SLC25A32 is widely differentially expressed in pan-cancer with prognostic significance and is correlated with immune infiltration. Additionally, it can affect breast cancer occurrence and development.

## 1. Introduction

Cancer prevalence and death are steadily rising, which is detrimental to societal advancement and human health [1]. Breast cancer is the most commonly diagnosed cancer. Therefore, improving timely diagnosis and comprehensive treatment should be prioritised [2]. Targeted therapy and immunotherapy have also gained popularity in tumour treatment recently. However, more appropriate prognostic biomarkers and therapeutic targets are needed to support their combination therapy [3].

The solute carrier transporter (SLC) family comprises over 300 membrane-bound proteins essential in regulating substrate transport exchange, drug absorption, and efflux [4]. SLC25A32 is located on chromosome 8q22.3 and contains seven exons [5]. It encodes the mitochondrial flavin adenine dinucleotide (FAD) transporter, an inner membrane carrier that imports FAD from the cytoplasm into the mitochondria. SLC25A32 dysfunction leads to mitochondrial FAD deficiency and impaired mitochondrial acyl-CoA dehydrogenase and electron transfer chain, further disrupting muscle and brain functions [6]. Studies on SLC25A32 have focused on neurological and metabolic diseases, where its mutations in humans lead to riboflavin-responsive motor intolerance [7]. However, earlier studies have suggested that SLC25A32 is a mitochondrial folate transporter protein [8]. SLC25A32 dysfunction disrupts folate-mediated one-carbon metabolism. Allelic alterations in SLC25A32 may lead to neural tube defects (NTD) in human fetuses, and SLC25A32 knockout mice may also develop neurological diseases in the embryonic period [9]. In addition, metabolic analysis suggests that disrupted SLC25A32 dysfunction is associated with multiple biochemical processes of ATP generation, including fatty acid *β* oxidation, catabolism of essential amino acids (leucine, isoleucine, valine, tryptophan, and lysine), and choline degradation [10]. SLC25A32 inhibition leads to respiratory chain dysfunction of FAD-dependent complex II enzymes, reactive oxygen species (ROS) induction, and reduced glutathione (GSH) depletion, which impairs cancer cell proliferation. Beyond the controversial substrate specificity of SLC25A32, its role in tumour progression is rarely discussed. Additionally, the pan-cancer systematic analysis and specific mechanism of action in the immune microenvironment need further characterisation and exploration.

## 2. Materials and Methods

### 2.1. Data Collection, Mining, and Processing

We obtained the mRNA expression profile of SLC25A32 and data related to prognosis, stage, DNA promoter methylation, copy number fragments, and clinical features of 33 cancers from The Cancer Genome Atlas (TCGA) (https://portal.gdc.cancer.gov/) database [11]. Ethics committee or institutional review board approval was not required because this was a bioinformatics study. In addition, special sources of data were annotated.

### 2.2. Transcriptional and Protein-Level Differential Expression Analysis of SLC25A32 in Pan-Carcinoma

First, at the transcriptional level, SLC25A32 expression in healthy and corresponding tumour tissues was collected using Tumour Immune Estimation Resource 2.0 (TIMER2.0) (http://timer.cistrome.org) [12], GEPIA2.0 (http://gepia2.cancer-pku.cn/#index) and UALCAN databases (http://ualcan.path.uab.edu/analysis-prot.html). Specific bioinformatics steps and processes can be referred to this article [13]. The GEPIA2.0 database simultaneously revealed the association between SLC25A32 and the pathological stages of different cancers [14]. Furthermore, the UALCAN database compared DNA promoter methylation levels of SLC25A32 in various cancer types [15], and RNA-seq data from TCGA were associated with methylation levels in UCSCXenaShiny (https://xena.ucsc.edu/) [16]. In addition, the difference in SLC25A32 protein expression levels was compared between the healthy and corresponding tumour tissues using the Clinical Proteomics Cancer Analysis Consortium (CPTAC) data in the UALCAN database, and the post-translational modification of SLC25A32 was studied using the PhosphoSitePlus database (https://www.phosphosite.org/) [17]. More detailed data analysis procedures for oncogene proteomics can be found in this article, including protein quantification and expression, as well as protein action pathway research methods [13].

### 2.3. Genetic and Mutational Situation Analysis of SLC25A32 in Pan-Carcinoma

Gene set cancer analysis (GSCA) (http://bioinfo.life.hust.edu.cn/GSCA/#/) is a database that aggregates information on tumour genomic gene sets [18]. We initially studied the copy number variation (CNV) of SLC25A32 in different cancers, cross-verified the correlation between SLC25A32 DNA methylation and transcriptional expression levels in different cancers via the GSCA database, and summarised gene expression and drug sensitivity using the Genomics of Drug Sensitivity in Cancer (GDSC) database (https://www.cancerrxgene.org/).

The correlation between SLC25A32 mutations, the site, copy number alteration (CNA), and the mRNA levels in SLC25A32 and CNV were analysed using the cBioPortal platform (https://www.cbioportal.org/) [19]. The detailed operation steps can be referred to this article [20]. We further analysed the relationship between SLC25A32, different CNA type groups, and pan-cancer tumour prognosis using the UCSCXenaShiny.

### 2.4. SLC25A32 Prognostic Analysis of Patients in Pan-Cancer

In computing the survival heat map for overall and disease-free survival (OS and DFS, respectively) in pan-cancer using the GEPIA2.0 database, *P*  < 0.05 was considered significant, and approaching red indicates the highly expressed SLC25A32 group in the corresponding cancer type with a worse prognosis, revealing multiple specific tumours. Furthermore, SLC25A32 OS, progression-free survival (PFS), and disease-specific survival (DSS) data in pan-cancer were visualised via a forest map using the UCSCXenaShiny platform.

### 2.5. Therapeutic Analysis of SLC25A32 Immune Infiltration in Pan-Carcinoma

Using the TIMER 2.0 database, multiple algorithms analysed the effect of SLC25A32 on the immune cell infiltration in 33 cancers [21]. Furthermore, we summarised the correlation between SLC25A32 expression, methylation, CNA conditions, and immunotherapy-related markers using the TISIDB database (http://cis.hku.hk/TISIDB/index.php) [22]. Specific correlation tests in different immune cells can be referred to this article, which includes detailed methods and instructions [13].

Using pan-cancer samples, stemness scores, tumour mutation burden (TMB), and microsatellite instability (MSI) scores from the TCGA database, the immunedeconv software package (version 2.0.3) revealed the relationship between tumour-infiltrating immune cells (TIC) and immunotherapy-related indicators and SLC25A32 gene expression data in pan-cancer [23].

### 2.6. SLC25A32 Function and Analysis of Biological Tumour Function at the Pan-Cancer Single-Cell Level

The CancerSEA platform (http://biocc.hrbmu.edu.cn/CancerSEA/), including various cancer single-cell sequencing data, can more accurately reveal the various biological cancer functions [24]. We analysed the relationship between SLC25A32 expression and various cancers' biological tumour behaviours through a t-SNE diagram that displays the SLC25A32 expression profile in several TCGA cancer single cells.

### 2.7. SLC25A32 Functional Enrichment Analysis and Co-Expressed Genes

The molecules that interact with SLC25A32 in pan-cancer were selected using BioGRID (https://thebiogrid.org/) [25]. In addition, the LinkedOmics database (http://www.linkedomics.orglogin.php) was also used to analyse the heat map of positive and negative gene co-expression with SLC25A32 in RNA-seq data of patients with breast cancer in TCGA [26]. Furthermore, Pearson's correlation coefficient was used to plot the volcano plot of co-expressed genes. Using the Metascape database (http://metascape.org/gp/index.html#/main/step1) for functional enrichment analysis of co-expressed genes in breast cancer [27], including gene ontology (GO) and the Kyoto Encyclopaedia of Genes and Genomes (KEGG) pathway.

The gene set enrichment analysis (GSEA) was performed using the clusterProfiler package for R (version 3.14.3) [28]. Enriched pathways were classified based on normalised enrichment scores and corrected *P*-values. The clusterProfiler package was similarly used for GO/KEGG analysis of the DEGs between breast cancer and healthy tissue samples [29, 30]. The results revealed that these genes exhibit multiple biological processes, including signalling pathways, molecular functions, and cellular components, which the ggplot2 package for R (version 3.3.3) annotated and visualised [31].

### 2.8. Protein–protein Interaction Network Analysis

Protein interaction network analysis was simultaneously performed using Metascape Online [27]. The results revealed a protein–protein interaction network comprising genes co-expressed with SLC25A32 in breast cancer, with different colour blocks demonstrating possible mechanisms of action affecting the tumour.

### 2.9. Cell Culture

Healthy mammary epithelial cell lines were cultured in an MCF-10A-specialised medium. Breast cancer cell lines, including MCF-7, MDA-MB-231, BT-549, and HCC1937, were obtained from the Cell Bank of the Chinese Academy of Sciences (Shanghai, China) and the Cancer Institute of Southern Medical University (Guangzhou, China) and grown in DMEM supplemented with 10% fetal bovine serum (Gibco, USA). All cells were grown at a constant temperature of 37°C in a 5% CO_2_ culture in the incubator.

### 2.10. Quantitative Real-Time Polymerase Chain Reaction (PCR)

The premix was tested on a CFX-96 real-time PCR instrument (Bio-Rad, USA). GAPDH was used as a normalisation control. The primers used are as follows: GAPDH-F: GGAGCGAGATCCCTCCAAAAT, GAPDH-R: GGCTGTTGTCATACTTCTCATGG, SLC25A32-F: TACGGGGACTTTATCAAGGAGT, and SLC25A32-R: AAGGCGAGTTTTTGTTACCCATA. We used the 2^−*ΔΔ*Ct^ method to quantitatively compare the cyclic threshold (Ct) value results.

### 2.11. Cell Transfection

Transfection was performed using the siRNA kit designed and synthesised by Guangzhou Ruibo Bio (Guangzhou, China). With transfection in six-well plates (NEST Biotechnology, China), cells in the exponential growth phase rose to 50%–60% using lipo3000 (Invitrogen Biotechnology, Shanghai, China). Cells were harvested for RNA extraction 24–48 hr post-transfection or functional experiments and at 72 hr for western blot analysis of extracted proteins.

### 2.12. Protein-Based Western Blot Analysis

The cell lysate was prepared using a 100 : 1 : 1 ratio of phosphatase inhibitor, protease inhibitor, and total protein extraction. The lysate was subjected to electrophoresis with 10% SDS-PAGE gel under pressure about 150 V, then transferred to PVDF at 350 mA, and blocked with 5% skimmed milk. The primary antibodies included: SLC25A32, E-ca, N-ca, vimentin, MMP 9, *β* -catenin, *β* -tubulin, and GAPDH.

### 2.13. Cell Proliferation Assay

Cells were harvested at 24–48 hr post-transfection, and digested cells were seeded into 96-well plates at a density of 2,000 cells per well. After 6 hr of apposition, 10 *μ*L of CCK-8 reagent was added per well, and absorbance was measured at 450 nm, with measuring time points at 0, 1, 24, 48, 72, and 96 hr. Three duplicate wells were set for each group. The experiments were repeated more than thrice.

### 2.14. Migration and Invasion Assays

Cells in six-well plates attained 100% confluence at 48 hr post-transfection. They were scratched using the pipette gun head, placed in PBS, and photographed at 0, 24, and 48 hr. The wound healing area was then determined. All assays were performed in triplicates, and representative images were selected.

Transwell (BD Biosciences, NJ, USA) assays were used to detect cell invasion abilities.

### 2.15. Statistical Analyses

Some statistical analyses and visualisation were performed using R (version 4.2.1), and public databases were analysed by default. Student's *t*-test was used to analyze the differences in gene expression, methylation level, and protein level in cancer. One-way ANOVA was used to analyze the pathological stage. Kaplan–Meier analysis and Log-rank test were used for survival analysis. Regarding breast cancer, the Wilcoxon rank sum and signed rank tests were used to detect the significance of SLC25A32 expression in unpaired and paired tissues, respectively. mRNA and protein levels, cell proliferation assays, and migration and invasion assays were analysed using Student's *t*-test. All tests in this study were two sided, and a *P*-value of <0.05 was considered significant. Due to the large number of databases and different statistical analysis methods involved in this paper, it should be alert that the false positive rate may increase, and more biological experimental validation is needed to confirm the results. The results of the statistical analysis have some limitations due to the differences between the number of samples and databases, and more samples are needed in the future to further supplement our conclusions.

## 3. Results

### 3.1. The Transcriptome Expression Level of SLC25A32 Varied in Most Cancers Compared with Healthy Samples

**Figure 1:**
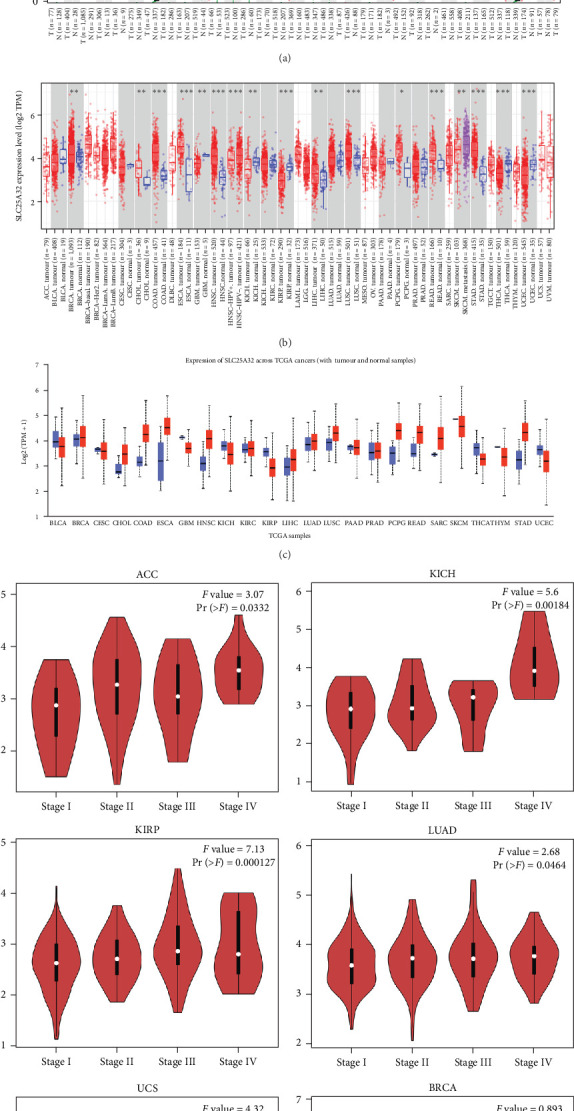
Multiple databases revealing the differential transcriptome map and pathological stage of SLC25A32 in pan-cancer. (a) The GEPIA2.0 platform revealed differential mRNA levels of SLC25A32 in paired tissues of pan-cancer. (b) The TIMER2.0 database uncovered the mRNA expression levels of SLC25A32 in unpaired cancer and healthy tissues. (c) The UALCAN platform showed the differential level of SLC25A32 in the pan-cancer at the mRNA level. (d) The GEPIA2.0 platform analysis presented a significant correlation between SLC2532 and the pathological stages of ACC, KICH, KIRP, LUAD, and USC tumours. *⁣*^*∗*^*P*  < 0.05; *⁣*^*∗∗*^*P*  < 0.01; and *⁣*^*∗∗∗*^*P*  < 0.001.

The differential expression of SLC25A32 in multiple tumours and corresponding healthy tissues at transcript levels was investigated using the TIMER2.0, GEPIA, and UALCAN databases. The GEPIA2.0 database presents the differential expression of SLC25A32 in paired tumours and healthy tissues, validating the evidence that SLC25A32 was significantly upregulated in most tumours, including lymphoid neoplasm diffuse large B-cell lymphoma (DLBC), oesophageal carcinoma (ESCA), pancreatic adenocarcinoma (PAAD), stomach adenocarcinoma (STAD), and thymoma (THTM) (Figure 1(a)). The unpaired expression analysis provided by TIMER2.0 revealed that SLC25A32 was significantly upregulated in 10 cancer types: breast invasive carcinoma (BRCA), cholangiocarcinoma (CHOL), colon adenocarcinoma (COAD), ESCA, head and neck squamous cell carcinoma (HNSC), liver hepatocellular carcinoma (LIHC), lung squamous cell carcinoma (LUSC), pheochromocytoma and paraganglioma (PCPG), rectum adenocarcinoma (READ), and STAD; downregulated in the five cancer types: glioblastoma multiforme (GEM), kidney chromophobe (KICH), kidney renal papillary cell carcinoma (KIRP), thyroid carcinoma (THCA), and uterine corpus endometrial carcinoma (UCEC) (Figure 1(b)). SLC25A32 expression analysis in the TCGA database revealed similar differential results (Figure 1(c)).

The GEPIA2.0 database revealed the differential expression of SLC25A32 in different pathological stages of various cancer types. SLC25A32 was significantly associated with the pathological stage of adrenocortical carcinoma (ACC), KICH, KIRP, lung adenocarcinoma (LUAD), and uterine carcinosarcoma (USC) (Figure 1(d)). In ACC and KICH, higher SLC25A32 transcript level expression predicted worse tumour stage and clinical manifestations. The cancer types without a significant effect of SLC25A32 expression on the disease pathology stage are presented in Figure S1.

### 3.2. SLC25A32 Promoter Methylation Differed across Multiple Cancer Types

**Figure 2:**
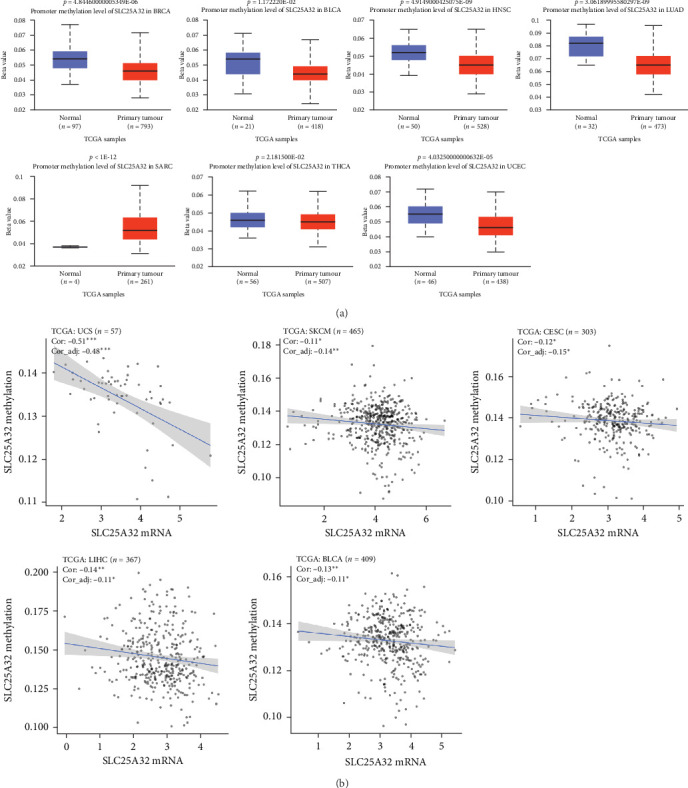
Differential promoter methylation levels of SLC25A32 in pan-cancer and the relationship with mRNA expression. (a) Abnormal promoter methylation levels of SLC25A32 in BLCA, HNSC, LUAD, SARC, THCA, and UCEC. (b) SLC2532 promoter methylation and transcriptome expression levels were related in UCS, SKCM, CESC, LIHC, and BLCA. *⁣*^*∗*^*P* < 0.05; *⁣*^*∗∗*^*P* < 0.01; *⁣*^*∗∗∗*^*P* < 0.001.

DNA methylation may lead to changes in the structure of chromosomes, which is closely related to the occurrence, development, and canceration of tumours. DNA methylation level and changes in specific gene methylation can be used as indicators for tumour diagnosis. Aberrant expression of oncogenes is usually correlated with DNA methylation levels [32]. Therefore, we investigated the promoter methylation level of SLC25A32 in pan-cancer. In BRCA, bladder urothelial Carcinoma (BLCA), HNSC, LUAD, Sarcoma (SARC), THCA, and UCEC SLC25A32 promoter methylation differed between normal tissues (Figure 2(a)). However, according to the TCGA database, a correlation exists between promoter methylation and transcriptome levels of SLC25A32 in UCS, SKCM, CESC, LIHC, and BLCA (Figure 2(b)). Thus, DNA promoter methylation might be a reason for the aberrant transcriptome expression of SLC25A32. The cancer types with no significant differences in SLC25A32 promoter methylation are presented in Figure S2, indicating that other factors cause transcriptomic level differences besides DNA promoter methylation abnormalities. We then analysed the genetic and epigenetic mutations of SLC25A32 in pan-cancer.

### 3.3. Genetic Alterations of SLC25A32 Affected Their Expression at the Transcriptomic Level and Correlated with Tumour Prognosis

Notably, we summarised the association between SLC25A32 expression and related drug sensitivity in various cancer types based on the anti-cancer drug susceptibility database Genomics of Drug Sensitivity in Cancer (GDSC). Cetuximab and Afatinib treatment sensitivity was positively correlated with SLC25A32 mRNA expression. In contrast, the treatment sensitivity of Dabrafenib and I-BET-762 demonstrated a negative correlation (Figure S2). The above results may suggest that clinicians could guide clinical medication according to the different SLC25A32 levels in different patients, and judge the sensitivity and tolerance of different patients to drugs. In addition, according to the correlation between treatment sensitivity and SLC25A32, it may develop new ideas for future targeted therapy.

**Figure 3:**
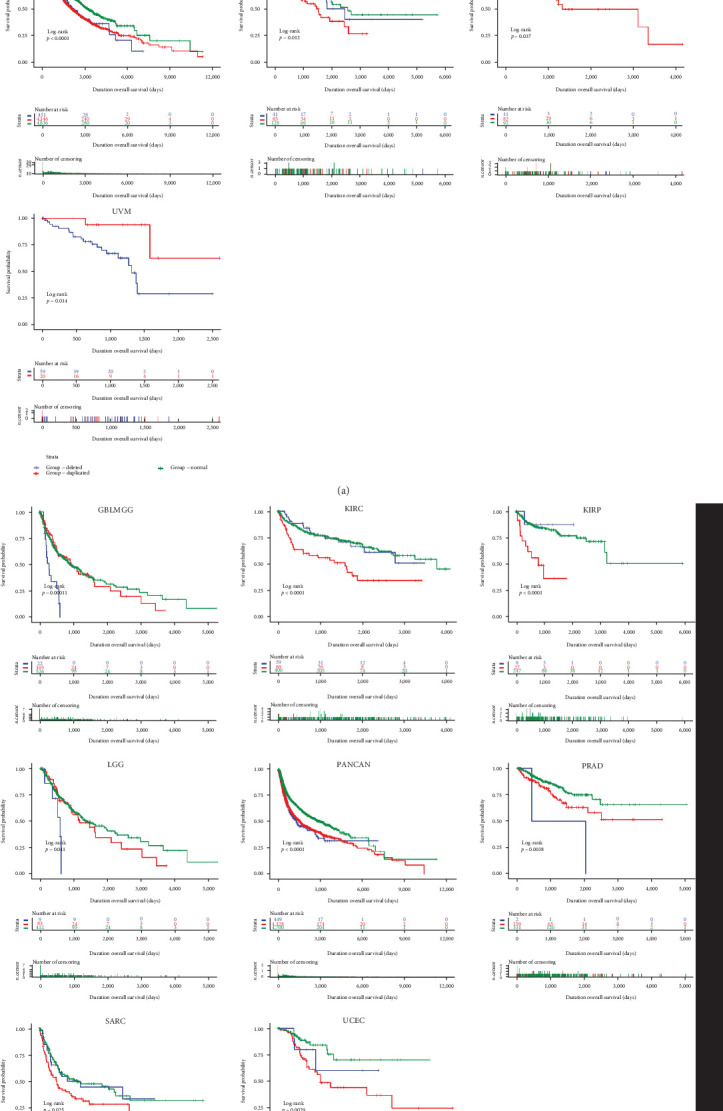
The different SLC25A32 CNA types cause the prognostic level differences of pan-cancer. (a) Correlation of the deleted and duplicated groups of SLC25A32 and the OS of GBLMGG, KIRC, KIRP, SARC, UCEC, UVM, and PANCAN. (b) Different CNA groups of SLC25A32 were associated with the PFS of GBLMGG, KIRC, KIRP, LGG, PRAD, SARC, UCEC, and PANCAN.

Using UCSCXenaShiny, we further explored the prognostic relationship of several CNA species in SLC25A32 and different cancer types, and in the total pan-cancer data, OS and PFS were lower in groups with deleted and duplicated SLC25A32 than in the normal group, and most significantly different tumour types. In conclusion, the above results can be initially reflected at the clinical level, in some cancer types, patients with SLC25A32 CNA pattern alterations had worse prognosis than those with normal SLC25A32. It is further suggested that CNA pattern of SLC25A32 may be used as an index to judge the malignant degree of tumour in clinical diagnosis and treatment. The level of CNA is associated with cancer mortality and recurrence, but the relationship between clinical outcome and the overall level of CNA contained in the tumour has not been fully studied, so we should be cautious in judging our conclusions. Moreover, the SLC25A32 duplicated group had more adverse OS and PFS prognosis data. In addition, the KIRC OS and KIRP PFS in the SLC25A32 normal group were between the SLC25A32 deletion and duplication groups (Figures 3(a) and 3(b)). TCGA pan-cancer data from the cBioPortal platform was used to study the genetic alterations of SLC25A32, and the gene copy number of SLC25A32 was analysed in pan-cancer. Data types on the cBioportal platform are stored at the gene level, analysed in conjunction with available deidentified clinical data, and then organised as a function of patients and genes. The main abstract concept is based on altered genes [19]. SLC25A32 was significantly amplified in most cancer types (Figure S3). Additionally, its gene amplification was significantly associated with the upregulation of mRNA expression (Figure S3). In addition to altered DNA promoter methylation in pan-cancer, genetic and epigenetic alterations are also involved in the upstream mechanism.

### 3.4. The Protein Expression Level of SLC25A32 Similarly Varied in the Pan-Cancer and Normal Samples

**Figure 4:**
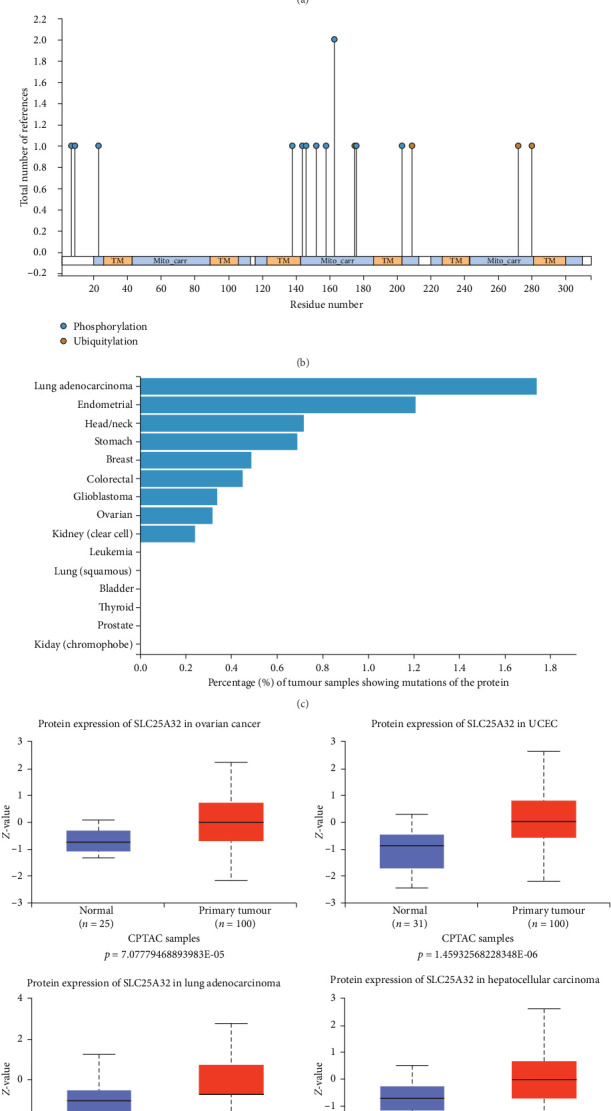
Proteomic differential analysis of SLC25A32 in pan-cancer. (a) The CPTAC database reveals differences in protein levels in multiple cancers and the corresponding healthy tissues. (b) The PhosphpSitePlus platform counted the types of post-translational modifications of SLC25A32 and the number of citations involved in previous studies. (c) Different tumour sample numbers reveal the frequency of SLC25A32 protein mutation in various cancer types. (d) The UALCAN database compares SLC25A32 protein expression individually in different tumours. *⁣*^*∗*^*P*  < 0.05; *⁣*^*∗∗*^*P*  < 0.01; *⁣*^*∗∗∗*^*P*  < 0.001.

Analysing the SLC25A32 protein expression level data from the CPTAC database in the UALCAN platform revealed that SLC25A32 protein expression increased over SLC25A32 in OV, UCEC, lung cancer, HNSC, and liver cancer and decreased over SLC25A32 in PAAD and glioblastoma (Figures 4(a) and 4(d)). To further analyse the SLC25A32 proteomics data, we investigated the possible post-translational modification sites of SLC25A32 and the mutation frequency in various tumour types using the PhosphpSitePlus database. Post-translational modifications in tumour cells mediate the activation of proto-oncogenes and the inhibition of tumour suppressor genes, weaken cell cycle regulation and enhance proliferation and growth signals, and promote the occurrence and rapid development of tumours. According to previous data, SLC25A32 mainly has two modification species: phosphorylation and ubiquitination (Figure 4(b)). Notably, we obtained mutation frequency data that are almost consistent with the UALCAN database because both have high SLC25A32 mutation frequency (Figure 4(c)) in lung adenocarcinoma, endometrial, HNSC, stomach, breast, colorectal, glioblastoma, and ovarian serous cystadenocarcinoma (OV). The proteomic data complement the transcriptome view that SLC25A32 has differential mRNA and protein expression in pan-cancer and healthy tissues and may be involved in tumour development. However, the regulation and specific mechanisms of SLC25A32 in tumour progression require further investigation.

### 3.5. SLC25A32 Has a Prognostic Value as a Novel Biomarker in Pan-Cancer

**Figure 5:**
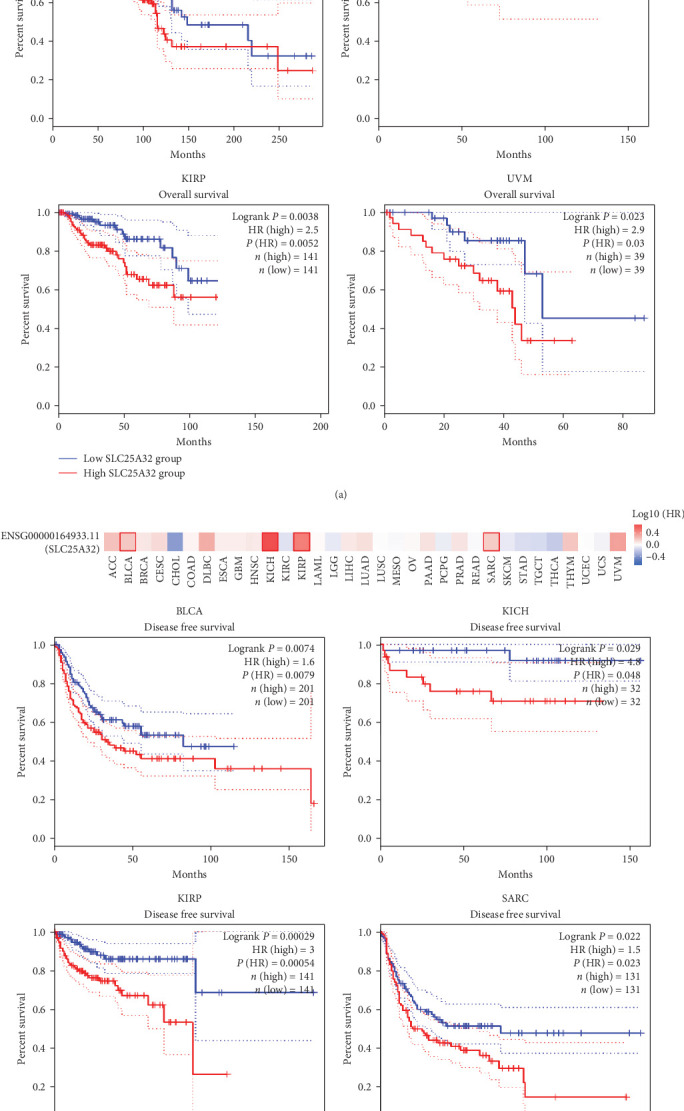
The prognostic value of SLC25A32 in pan-cancer using the survival heat map and KM curve. (a) The GEPIA2.0 database, using calculation of survival heat map and KM curves in each tumour, revealed that the high and low SLC25A32 expressions were closely associated with OS levels in BLCA, BRCA, HNSC, KICH, KIRP, LAML, LUAD, MESO, SARC, and UVM. (b) Using a similar method for analysis, the group with high SLC25A32 expression had a worse DFS in patients with BLCA, KICH, KIRP, and SARC. *P*  < 0.05, and |logFC | > 1 indicated significant differential prognostic data.

Using the GEPIA database to investigate the prognostic significance of SLC25A32 in multiple cancer types, OS and DFS map of SLC25A32 revealed that patients with lower class tumours that highly express SLC25A32 have more adverse OS, including BLCA, BRCA, HNSC, KICH, KIRP, LAML, LUAD, MESO, SARC, and UVM (Figure 5(a)). The GEPIA database is built from HTML5 and JavaScript libraries with a variety of basic algorithmic principles and methods for analysing gene expression. For example, expression-based clustering can be divided into supervised and unsupervised methods [14]. In addition, high SLC25A32 expression was associated with poor DFS data in patients with BLCA, KICH, KIRP, and SARC (Figure 5(b)). Furthermore, the OS, progressive-free interval (PFI), and disease-specific survival (DSS) data of SLC25A32 in different cancer types were analysed using the UCSCXenaShiny database (Figure S4). Both results indicate that SLC25A32 is significantly associated with prognosis in most cancer types and is a risk factor for cancer prognosis.

### 3.6. SLC25A32 Expression Is Closely Associated with the Tumour Immune Microenvironment and Immunotherapy

**Figure 6:**
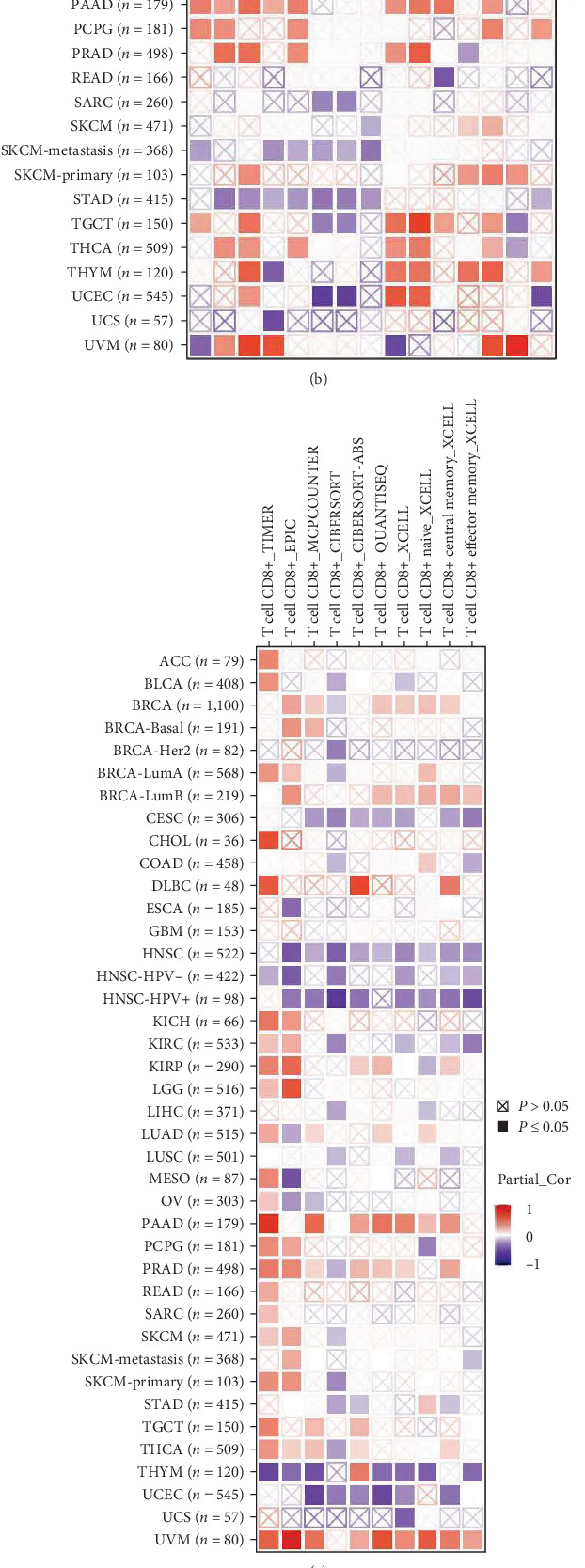
SLC25A32 immune infiltration analysis in pan-carcinoma. (a) TIMER2.0 database analysis determined the relationship between SLC25A32 expression and immune infiltration of tumour-associated fibroblasts in pan-cancer. (b) A similar approach was used to analyse the relationship between SLC25A32 expression and B-cell immune infiltration in pan-carcinoma. (c) The relationship between SLC25A32 expression and immune infiltration of CD8 T cells in pan-cancer was analysed. (d) The relationships between SLC25A32 expression and CD4 T cells, Th 1, and Th 2 immune infiltration in pan-cancer were analysed. (e) Correlation analysis of integrated SLC25A32 expression with 20 immune-infiltrating cells in pan-carcinoma using UCSCXenaShiny. (f) Radar plot of the correlation between mRNA SLC25A32 expression and different levels of tumour stemness. (g, h) Radar plots displaying the correlation of SLC25A32 expression with TMB/MSI in pan-cancer. *⁣*^*∗*^*P*  < 0.05; *⁣*^*∗∗*^*P*  < 0.01; *⁣*^*∗∗∗*^*P*  < 0.001.

In the immune microenvironment, immune infiltration is closely related to tumour progression [33]. The TIMER database mainly completed the analysis by using the R package integrated with six state-of-the-art algorithms, including xCell, MCP-counter, EPIC and quanTIseq, capable of allowing users to study Spearman correlations between genes of interest and immune cell types in the gene module. In addition, at the algorithmic level, the algorithms used to calculate immune infiltration can be divided into two categories: genetic feature-based and deconvolution methods [12]. Thus, we calculated the correlation between SLC25A32 expression and different immune cell infiltration in multiple cancer types. Tumour-associated fibroblast infiltration was positively correlated with SLC25A32 expression in CHOL, HNSC, KIRP, MESO, thymoma (THYM), UCS, and PAAD. Contrastingly, a negative correlation was observed with SLC25A32 expression in DLBC and brain lower grade gliom (LGG) (Figure 6(a)). A heatmap of B-cell infiltration revealed that B-cell infiltration was positively correlated with SLC25A32 expression in CHOL, LGG, ACC, DLBC, KICH, PAAD, and PCPG and negatively correlated in CESC, HNSC, LUSC, and STAD (Figure 6(b)). Similarly, CD8 T cell infiltration was positively correlated with SLC25A32 expression in DLBC, PAAD, and UVM; however, it was negatively correlated with SLC25A32 expression in CESC, HNSC, and UCEC (Figure 6(c)). Notably, CD4 T cell infiltration determined using the XECLL algorithm revealed that Th1 cell infiltration was negatively correlated with SLC25A32 expression in almost all cancer types. In contrast, Th2 cell infiltration was positively correlated with SLC25A32 expression. This indicated that the Th1/Th2 ratio was significantly negatively correlated with SLC25A32 expression in almost all cancer types (Figure 6(d)). In addition, the correlation analysis between SLC25A32 expression and 20 kinds of immune infiltrating cells in pan-cancer revealed that SLC25A32 expression was negatively correlated with Treg cells, CD8 T cells, plasma cells, natural killer (NK) activated cells, mast resting cells, and B cell memory in pan-cancer. A significant positive correlation was observed with CD4 T cell memory, mast activated, M1 and M0 macrophages, and B cell naïve (Figure 6(e)).

Therefore, SLC25A32 expression is closely associated with immune cell infiltration in various cancer types. Additionally, it may serve as a novel immune-related biomarker in tumorigenesis and progression. Subsequently, we investigated the relationship between SLC25A32 expression, methylation, and CNA levels and three immunomodulators (Figure S5). It should be emphasised that we focused on the relationship between the above three research objects and SLC25A32 expression, and the conclusions among the three may be correlated, but there is no causal relationship, and no causal inference can be made through our research results.

We then utilised the relationship between SLC25A32 expression and TMB and MSI in most cancer types to investigate the relationship between SLC25A32 expression and immunotherapy. TMB and MSI are critical in deciding whether to proceed with immune checkpoint therapy [34]. Our findings revealed that SLC25A32 expression was positively correlated with TMB in some cancers, including DLBC, KICH, LUAD, and STAD. However, a significant negative correlation was lacking between SLC25A32 expression and TMB in UVM, KIRP, and READ (Figure 6(f)). SLC25A32 expression level was positively correlated with MSI in most cancers, such as UCEC, SKCM, LUAD, and glioblastoma multiforme (GBM) (Figure 6(g)). Tumour stemness is closely associated with the development of drug resistance and tumour cell proliferation during treatment [35]. Therefore, we extracted the correlation between SLC25A32 expression and stemness scores of different tumour types.  Figure 6 reveals a significant positive correlation between stemness scores and SLC25A32 expression in most tumours, including STAD, LAML, COAD, ESCA, and STAD. LUSC, LUAD, BLCA, and LGG; stemness scores were negatively correlated with SLC25A32 expression in some tumours, such as THYM, CHOL, and KICH (Figure 6(h)). These findings suggest that SLC25A32 may serve as a novel immune-related biomarker for tumour development and may provide new ideas for targeted immunotherapy.

### 3.7. SLC25A32 Expression at the Single-Cell Level Is Closely Associated with Biological Tumour Behaviour

**Figure 7:**
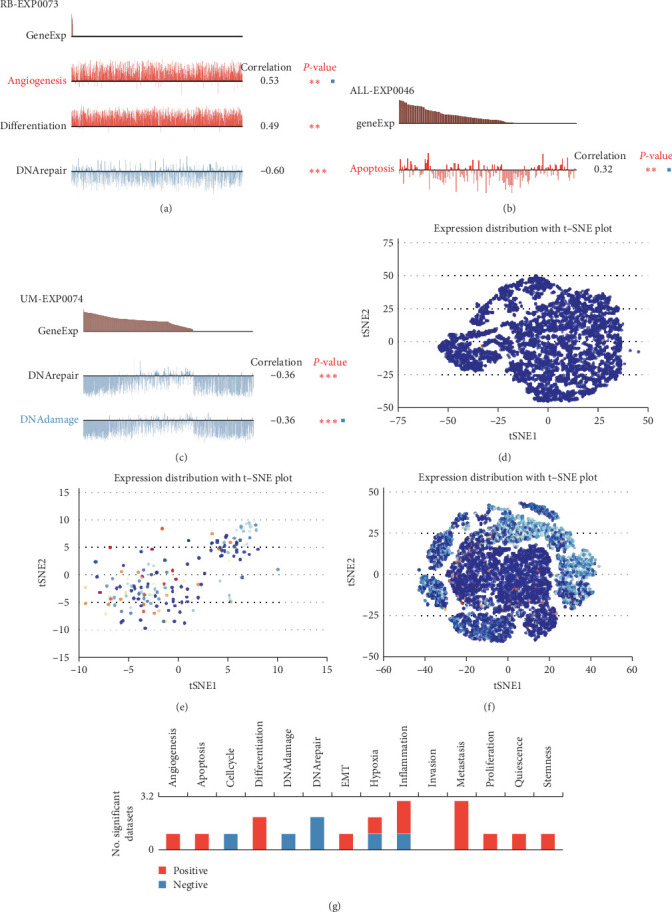
Biological functional analysis of SLC25A32 in pan-cancer at the single-cell level. (a–c) Functional analysis of SLC25A32 with individual cancer species cell datasets using the CancerSEA platform, including RB-EXP0073, ALL-EXP0046, and UM-EXP0074. (d–f) The expression profile of SLC25A32 at the single cell level in RB, three tumour types were visualised in t-ALL and UM using t-SNE plots. (g) The relationship between various tumour-related biological behaviours and functions in pan-cancer; red and blue indicate a positive and negative correlation, respectively, *⁣*^*∗*^*P*  < 0.05; *⁣*^*∗∗*^*P*  < 0.01; *⁣*^*∗*^*⁣*^*∗*^*⁣*^*∗*^*P*  < 0.001.

We analysed the correlation data using the CancerSEA database to explore the relationship between SLC25A32 expression at the single-cell level and the biological behaviour and function of most tumours. SLC25A32 expression is positively correlated with the malignant biological functions of most tumours, including metastasis, differentiation, inflammation, angiogenesis, apoptosis, cell proliferation, stemness, and epithelial–mesenchymal transition (EMT). However, a significant negative correlation was observed with the biological functions of cellular DNA damage repair and cell cycle (Figure 7(g)). In addition, we explored the correlation of SLC25A32 with biological behaviours and functions in single-cell datasets of different cancers. SLC25A32 significantly promoted angiogenesis and differentiation but inhibited DNA repair in the RB-EXP0073 dataset (Figure 7(a)). In the ALL-EXP0046 dataset, it was positively correlated with apoptosis (Figure 7(b)). A negative correlation was observed with DNA damage and repair in UM-EXP0074 single-cell data (Figure 7(c)). Moreover, SLC25A32 expression at the single-cell level was visualised using t-SNE plots (Figure 7(d)–7(f)). In conclusion, SLC25A32 expression may be closely associated with the malignant biological functions of most cancer types, promoting tumour cell metastasis and proliferation and inhibiting DNA damage repair. For clinical-level applications, it can be suggested that patients with high SLC25A32 expression may have a greater chance of tumour metastasis and other malignant manifestations.

Since mRNA molecules in the droplets are not captured by the magnetic beads in the same ratio, specificity bias may be caused, and it becomes the main cause of data sparsity. The main methods used to deal with data sparsity are normalisation and scaling techniques [36]. Alternatively, batch effects may be caused by unavoidable technical differences, such as the number of repeated freeze–thaw of samples, differences in methods of RNA extraction, sequencing depth, etc. The recommended way to handle batch effects is to record the batch information of the sample, subtract this effect in the downstream regression analysis, and strive to keep these variables constant during the experimental operation, or to avoid batch effects by combining cells from different experimental conditions and samples for subsequent operations [37]. Regarding the issue of technical artifacts and noise, the current CellBender model can model drop-based single-cell detection and eliminate systematic background noise, thereby enhancing biosignals and improving downstream analysis [38].

### 3.8. Co-Expression Genes, Functional Enrichment, and Protein–protein Interaction Network Analysis of SLC25A32 in Breast Cancer

**Figure 8:**
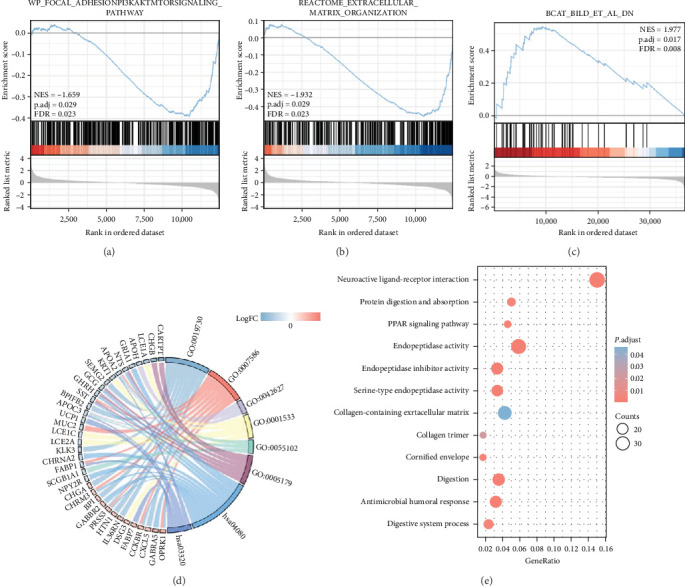
SLC25A32 functional enrichment analysis in breast cancer. (a–c) GSEA enrichment analysis revealed significant enrichment of SLC25A32 in WP_FOCAL_ADHESIONPI3KAKTMTORSIGNALING_PATHWAY, REACTOME_EXTRACELLULAR_MATRIX_ORGANIZATION, and BCAT_BILD_ET_AL_DN pathway in breast cancer. (d) String plots reveal the combined logFC analysis of GO/KEGG. (e) Bubble plots presenting the GO/KEGG enriched pathways of SLC25A32 in breast cancer.

Evaluation of the genes co-expressed with SLC25A32 revealed the possible mechanism of SLC25A32 in tumour progression (Figure S6). DCAF13 was strongly correlated with SLC25A32 in most cancers (*R* = 0.75), followed by FAM91A1 (*R* = 0.69) and TAF2 (*R* = 0.68). Thereafter, we investigated the specific regulatory mechanism of SLC25A32 in breast cancer progression. Enrichment analysis was used to evaluate the molecular mechanism of SLC25A32 action. GSEA revealed that SLC25A32 was enriched in PI3KAKTMTORSIGNALING _PATHWAY and EXTRACELcLULAR_ MATRIX_ ORGANIZATION pathways in breast cancer (Figures 8(a) and 8(b)). Recent studies have shown that SLC25A32 can activate the PI3K/AKT signalling pathway, leading to malignant proliferation and invasion of GBM cells [39]. Previous studies revealed that in addition to the structural role, the extracellular matrix also affects cell behaviour, such as proliferation, adhesion, and migration [40]. Furthermore, SLC25A32 was enriched in the *β*-catenin repressor gene marker (BCAT_BILD_ET_AL_DN), suggesting that SLC25A32 might regulate *β*-catenin activity (Figure 8(c)). In addition, string and bubble plots visualised the GO/KEGG analysis and revealed other functional roles of SLC25A32 in breast cancer (Figures 8(d) and 8(e)). The LinkedOmics platform and Metascape were used to further analyse the gene and functional enrichment of SLC25A32 co-expressed in breast cancer (Figure S7).

### 3.9. SLC25A32 Promoted the Proliferation, Invasion, and Migration of Breast Cancer Cells

**Figure 9:**
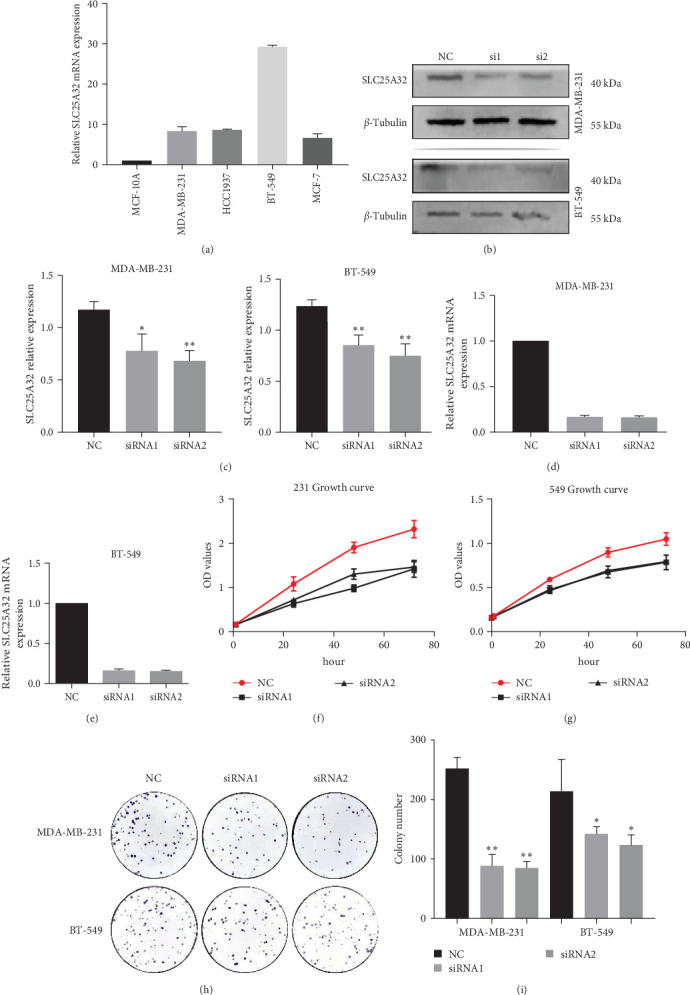
SLC25A32 promotes the proliferation, invasion, and migration of breast cancer. (a) Quantitative RT-PCR results comparing SLC25A32 expression levels in healthy breast epithelium and four breast cancer cell lines. (b) The 231 and 549 cell lines were selected for subsequent experiments, and SLC25A32 expression levels were verified using western blotting after knocking down both fragments. The blots were cropped from the full-length original blot to improve the clarity and conciseness of the presentation. (c) Grey value ratio of target/reference protein in 231 and 549 cell lines. (d, e) Changes in SLC25A32 mRNA levels post-transfection in 231 and 549 cells were verified using PCR. (f, g) CCK-8 assay was used to detect the proliferation of 231 and 549 cells post-transfection. Plate cloning assay was used to detect the colony-forming ability of the two cell lines post-transfection. (h, i) Plate cloning assay was used to detect the colony-forming ability of the two cell lines post-transfection. *⁣*^*∗*^*P*  < 0.05; *⁣*^*∗∗*^*P*  < 0.01.

**Figure 10:**
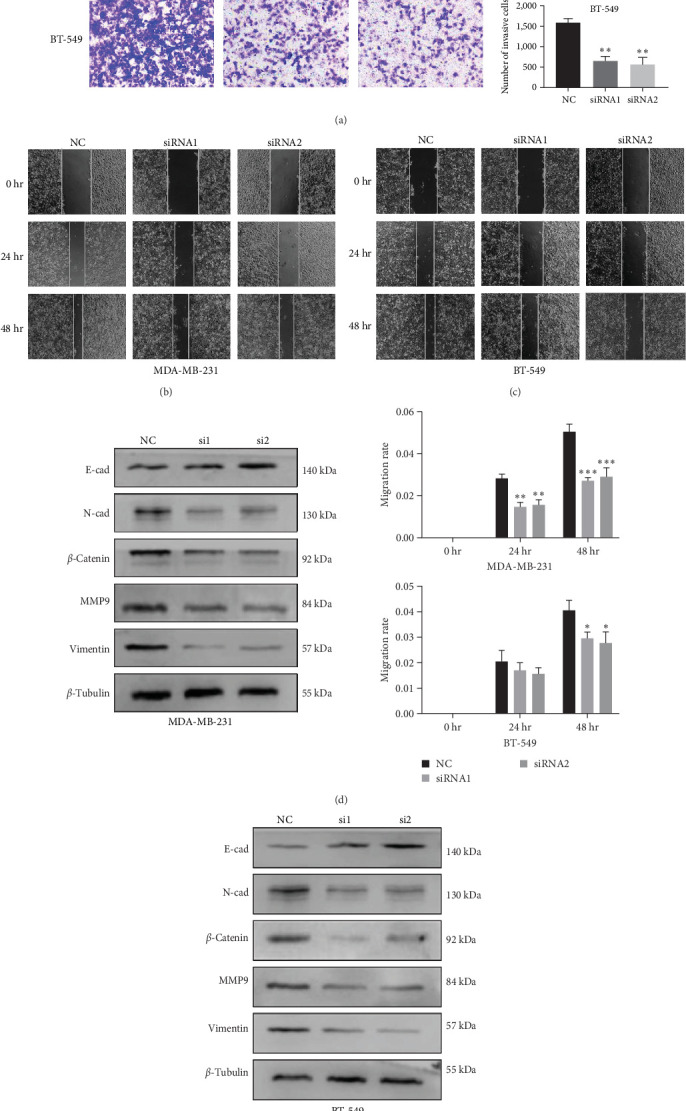
SLC25A32 promotes EMT in breast cancer. (a) Transwell assays were used to detect the invasion of the two groups of cells. (b) The migration ability of 231 cell lines was detected using scratch tests at 0, 24, and 48 hr, respectively. The migration rate at a certain time = (0 hr scratch area − scratch area at a certain time)/0 hr scratch area × 100%. (c) The migration ability of 549 cells was determined using the same method, and the bar graph illustrates the migration rate. (d, e) Western blot was used to detect the expression changes of EMT marker proteins in 231 and 549 cell lines after transient SLC25A32 knockdown. The blots were cropped from the full-length original blot to improve the clarity and conciseness of the presentation. *⁣*^*∗*^*P* < 0.05; *⁣*^*∗∗*^*P* < 0.01; *⁣*^*∗∗∗*^*P* < 0.001.

Bioinformatics analysis revealed the role of SLC25A32 in breast cancer, and we further explored its biological function. PCR confirmed that SLC25A32 expression was relatively highest in MDA-MB-231 and BT-549 cells; therefore, we selected both cell lines for subsequent experiments (Figure 9(a)). The two siRNA knockdown vectors were transfected into 231 and 549 cells, and the knockdown efficiency was verified using RT-PCR and western blotting. The two fragments could be successfully transfected in 231 and 549 cells (Figure 9(b)–9(e)). The effect of SLC25A32 on cell proliferation was determined using the CCK-8 assay. SLC25A32 knockdown by both fragments significantly reduced the proliferation ability of 231 and 549 cells (Figures 9(f) and 9(g). Plate cloning experiments demonstrated similar results (Figures 9(h) and 9(i)). Subsequently, the migration and invasion abilities of the 231 and 549 cells was reduced when SLC25A32 was downregulated in triple-negative breast cancer (TNBC) cells (Figure 10(a)–10(c)), indicating that SLC25A32 can affect the biological functions of TNBC cells proliferation and migration.

### 3.10. SLC25A32 Promoted EMT of Breast Cancer Cells

Based on biological behaviours and functions at the single-cell level, SLC25A32 may positively correlate with malignant biological behaviours, such as metastasis, tumour differentiation, angiogenesis, and EMT. Moreover, enrichment analysis revealed that SLC25A32 was enriched in PI3K/AKT/mTOR and *β*-catenin signalling-associated pathways and extracellular matrix. We hypothesised that SLC25A32 might similarly affect tumour cell metastasis and EMT in TNBC cells. EMT is involved in the malignant progression of tumours; therefore, we examined the changes in EMT-associated indicators in transfected TNBC cells. Western blotting revealed that EMT marker protein expression was significantly changed after the SLC25A32 knockdown. N-cadherin, *β*-catenin, vimentin, and MMP9 expressions were significantly downregulated in both TNBC cells, while E-cadherin expression was increased (Figures 10(d) and 10(e)). Therefore, SLC25A32 promoted the invasion, metastasis, and EMT of TNBC cells.

## 4. Discussion

The mitochondrial carrier family SLC25, one of the solute carrier transporters embedded in its inner membrane, is ubiquitous in eukaryotes. It transports multiple compounds in the mitochondrial inner membrane, participates in multiple metabolic pathways, and regulates cellular functions as a pathway connecting the mitochondrial matrix and cytoplasm [41, 42]. However, studies on the role of its family member SLC25A32 in malignancy are insufficient. Here, a systematic bioinformatics analysis combined with experiments can validate SLC25A32 as an innovative pan-cancer prognostic and immune-related biomarker and reveal its targetable mechanism of action in breast cancer. Folate metabolism is regulated by SLC25A32 in Chinese hamster ovary cells [43], and monocarbon and folate metabolism have been previously linked to tumour growth [44]. A novel data from a genetic mutant mouse model suggest that SLC25A32 dysfunction leads to FAD deficiency, secondary to defects in folate metabolism [45]. Another recent study also shows that the knockdown of SLC25A32 resulted in decreased mitochondrial flavin content and affected the stability and function of respiratory complex I [46]. Mitochondrial function is critical to cancer biology, and our conclusion that knockdown of SLC25A32 impairs breast cancer cell proliferation is consistent with the above conclusion. This may indicate that SLC25A32, which is highly expressed in cancer, acts as a mitochondrial FAD transporter and responds to the high level of mitochondrial oxidative phosphorylation in cancer cells to provide energy for the rapid proliferation of cancer cells and improve the anti-oxidative stress ability of tumour.

Thus, SLC25A32 might participate in carcinogenesis and the growth of most malignancies.

Epigenetic abnormalities may alter gene expression levels. Simultaneously, targeting epigenetic regulation to reprogramme cancer to a healthy state has gained increasing attention [47]. The correlation suggests that promoter methylation may cause the altered SLC25A32 expression in some cancers. Genetic alteration affects gene expression [48]. Therefore, we analysed the gene mutations of SLC25A32 and CNV in pan-cancer. In most cancers, SLC25A32 CNV types are mainly gene amplifications, and CNV alterations exhibit some correlation with their expression level. These results indicate that SLC25A32 alteration at the genetic level may also affect tumour growth and prognosis. Thus, the specific mechanism of CNV action on pan-cancer prognosis requires further studies. It should be noted that there is great intertumoral heterogeneity among different tumours, and the application of results for SLC25A32 needs to be rigorous and limited. For example, SLC25A32 had higher mRNA and protein levels in most cancers, indicating a worse prognosis, but in a few specific cancers, SLC25A32 expression was actually lower than that in normal tissues.

Evidence exists that genes influence tumour growth and prognosis by changing the immune microenvironment and cell infiltration [49]. Tumour-infiltrating CD8+ T cells demonstrated anti-tumor activity in previous studies and had favourable effects on the survival of patients with breast cancer [50]. CD4+ T cells have two subsets—Th 1 and Th 2 cells—and some clinical data observed unbalanced Th 1/Th 2 levels in patients with breast cancer, where Th 2 cells released the cytokines IL-4 and IL-10 and suppressed the host immune system, exhibiting a tumour-promoting effect [51]. In contrast, Th 1 cell infiltration resulted in a better prognosis and lower recurrence rate [52]. Thus, SLC25A32 might influence tumour progression by regulating immune cell infiltration and function. Immune-related markers are crucial in tumour immunity [53]. Hence, we investigated the relationship of mRNA expression, methylation, and CNA levels of SLC25A32 with the three TISIDB immune markers. There may be correlation among the three conclusions, but there is no causal link. Our findings suggest that the correlation between SLC25A32 methylation, CNV levels, and immune-related markers in some cancers provides new ideas for tumour immunotherapy response regulation.

This study has limitations. First, the part of bioinformatics analysis that only uses cross-analysis of multiple public databases, despite multiple crossovers and repeated alignments, and did not use clinical samples specifically collected for sequencing analysis, making our data more targeted. Second, despite conducting multiple functional experiments to verify the possible role and mechanism of SLC25A32 in breast cancer, *in vitro* experiments and immune infiltration of SLC25A32 and immunotherapy are lacking. Moreover, the regulatory mechanism of SLC25A32 in specific signalling pathways remains unclear. Our integrative analysis and experimental validation provide insight for future studies. In addition, although we have conducted bioinformatics studies on a variety of pan-cancers, our experimental data are only limited to breast cancer, and the inference of the experimental results of SLC25A32 to pan-cancers is limited, and more experiments are needed to verify it in the future.

It is worth noting that the consideration of tumour heterogeneity should be emphasised because this article involves multiomics data between many different cancers and experimental validation in a single cancer. Tumour heterogeneity includes both intratumor and intertumoral heterogeneity, and we have minimised the effect of reducing intratumor heterogeneity by adding experimental tumour cell lines and increasing the number of experimental replicates. Meanwhile, in the future, we expect to analyze multiomics data and calculate Jaccard similarity coefficient to explore the level of heterogeneity of SLC25A32 between tumours and patients to increase the clinical translation prospects in different tumours.

Overall, this study integrated expression, genetics, prognosis, immunity, and functional perspectives of SLC25A32, a novel pan-cancer prognosis gene, and immunotherapy-related biomarkers. We preliminarily demonstrated the influence and underlying mechanism of SLC25A32 on biological tumour behaviour in breast cancer via functional and pathway experiments on proliferation, invasion, and migration.

## Data Availability

Data supporting this reasearch article are available on request.

## Supplementary Materials

Supplementary Materials The Supplementary Material includes all the supplementary pictures cited in the article: Figure S1: shows that the cancer types without a significant effect of SLC25A32 expression on the disease pathology stage. Figure S2: shows the effect of copy number variations (CNV) on SLC25A32 transcript expression, the percentage of heterozygous/homozygous CNV (deletion/amplification) status in multiple cancer types using the GSCA database. Figure S3: shows that SLC25A32 was significantly amplified in most cancer types. Figure S4: shows the OS, progressive-free interval (PFI), and disease-specific survival (DSS) data of SLC25A32 in different cancer types using the UCSCXenaShiny database. Figure S5: shows the relationship between SLC25A32 expression, methylation, and CNA levels and three immunomodulators. Figure S6: shows that evaluation of the genes co-expressed with SLC25A32 revealed the possible mechanism of SLC25A32 in tumour progression. Figure S7: shows that the LinkedOmics platform and Metascape were used to further analyse the gene and functional enrichment of SLC25A32 co-expressed in breast cancer. Raw data includes all raw data and image data to verify the rationality of this paper.
