# Supplementary material for: Mitochondria-Associated Gene SLC25A32 as a Novel Prognostic and Immunotherapy Biomarker: From Pan-Cancer Multiomics Analysis to Breast Cancer Validation
Source: Anal Cell Pathol (Amst). 2024 Apr 29;2024:1373659. doi: 10.1155/2024/1373659 (PMC11611429; doi:10.1155/2024/1373659)
Supplement: Supplementary Materials — The Supplementary Material includes all the supplementary pictures cited in the article: Figure S1: shows that the cancer types without a significant effect of SLC25A32 expression on the disease pathology stage. Figure S2: shows the effect of copy number variations (CNV) on SLC25A32 transcript expression, the percentage of heterozygous/homozygous CNV (deletion/amplification) status in multiple cancer types using the GSCA database. Figure S3: shows that SLC25A32 was significantly amplified in most cancer types. Figure S4: shows the OS, progressive-free interval (PFI), and disease-specific survival (DSS) data of SLC25A32 in different cancer types using the UCSCXenaShiny database. Figure S5: shows the relationship between SLC25A32 expression, methylation, and CNA levels and three immunomodulators. Figure S6: shows that evaluation of the genes co-expressed with SLC25A32 revealed the possible mechanism of SLC25A32 in tumour progression. Figure S7: shows that the LinkedOmics platform and Metascape were used to further analyse the gene and functional enrichment of SLC25A32 co-expressed in breast cancer. Raw data includes all raw data and image data to verify the rationality of this paper. [file 1373659.f1.doc]

### Supplementary Material

Article title: Mitochondria-associated gene SLC25A32 as a novel prognostic and immunotherapy biomarker: From pan-cancer multi-omics analysis to breast cancer validation

E-mail address of the corresponding author: tangyao0626@126.com

**Supplementary figure S1. The cancer types without a significant effect of SLC25A32 expression on the disease pathology stage are presented.**


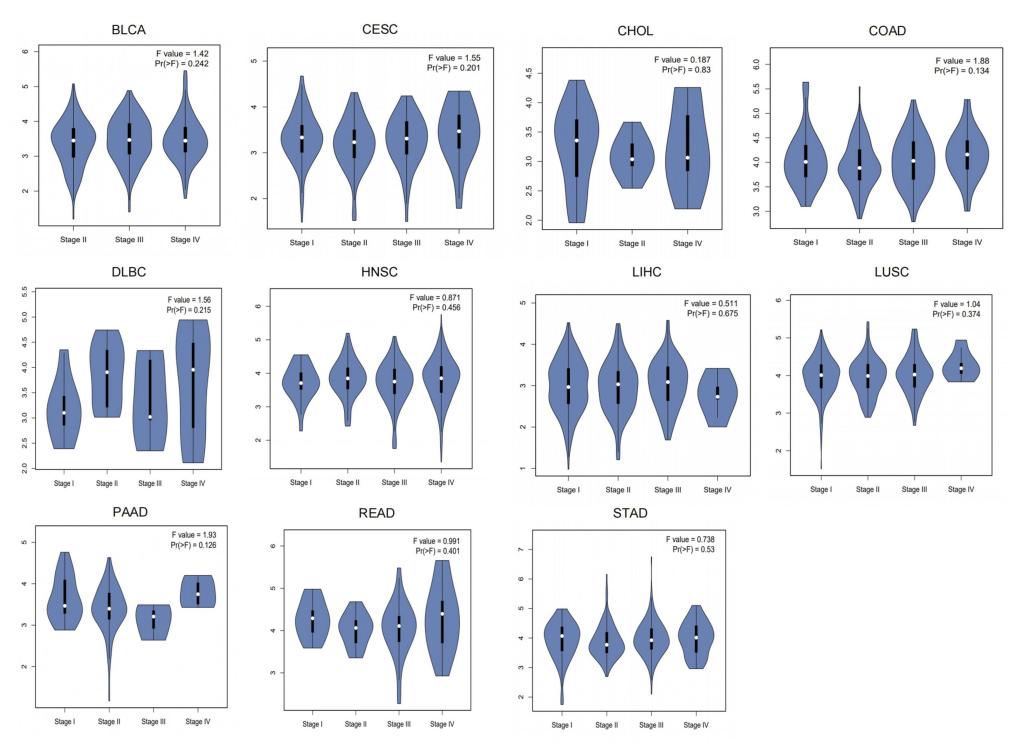


**Supplementary figure S2. The effect of copy number variations (CNV) on SLC25A32 transcript expression, the percentage of heterozygous/homozygous CNV (deletion/amplification) status in multiple cancer types using the GSCA database.**


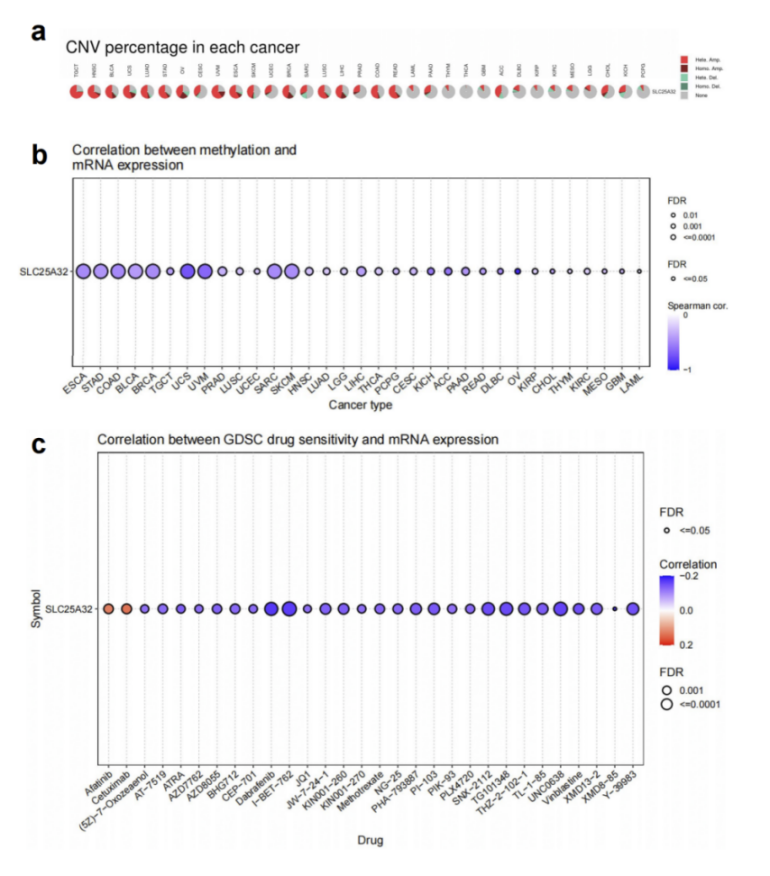


**Supplementary figure S3. SLC25A32 was significantly amplified in most cancer types.**


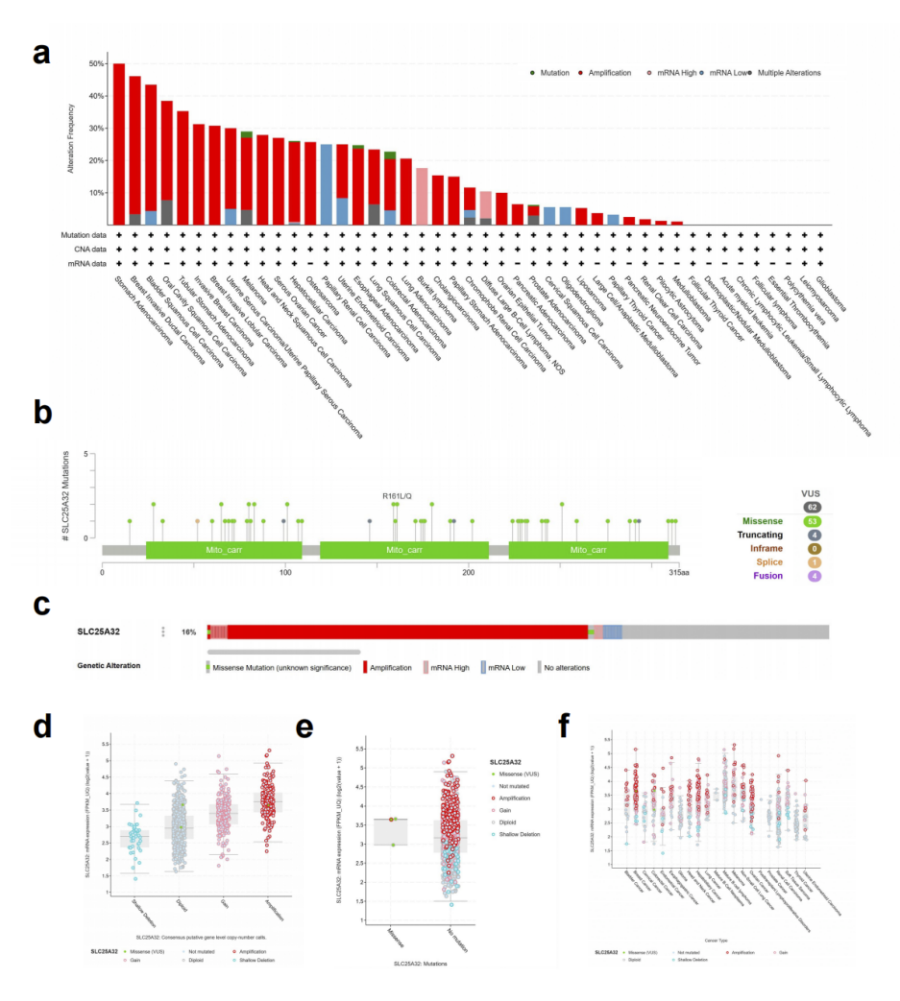


**Supplementary figure S4.The OS, progressive-free interval (PFI), and disease-specific survival (DSS) data of SLC25A32 in different cancer types were analysed using the UCSCXenaShiny database.**


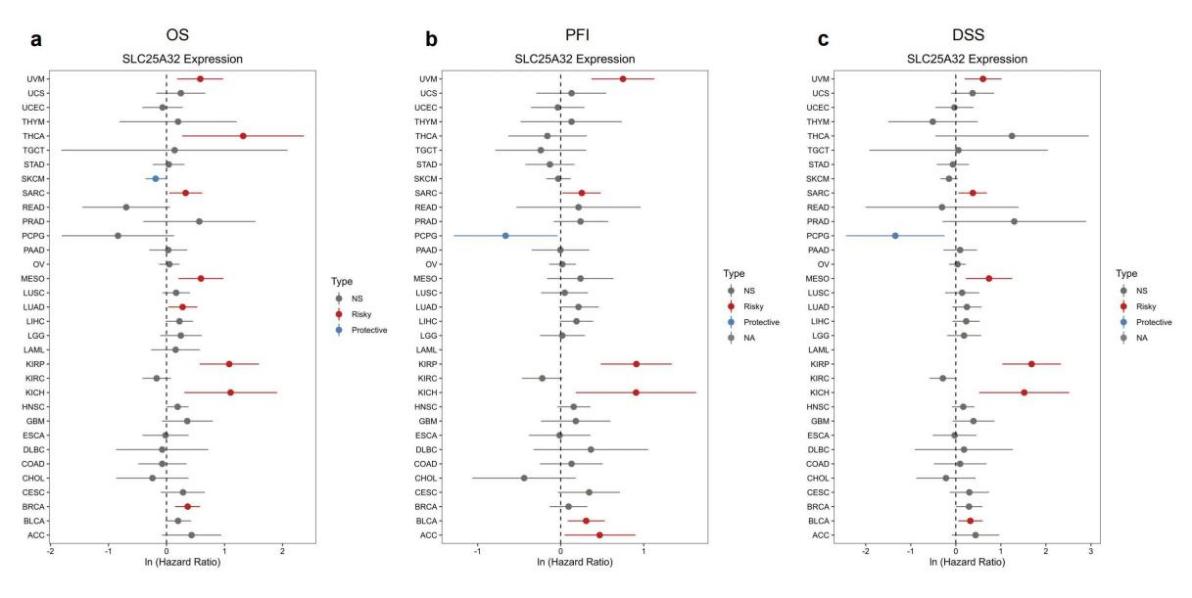


**Supplementary figure S5. The relationship between SLC25A32 expression, methylation, and CNA levels and three immunomodulators.**

**
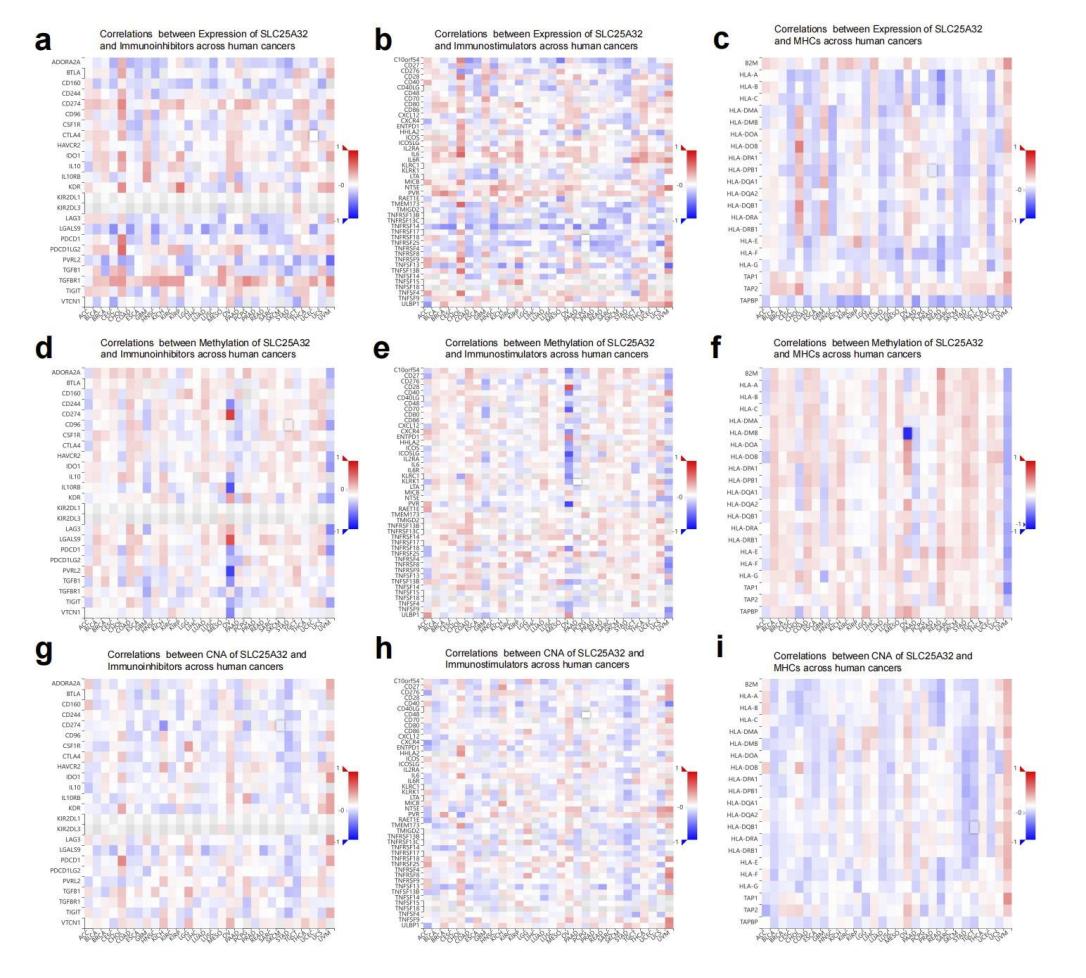
**

**Supplementary figure S6. Evaluation of the genes co-expressed with SLC25A32 revealed the possible mechanism of SLC25A32 in tumour progression**


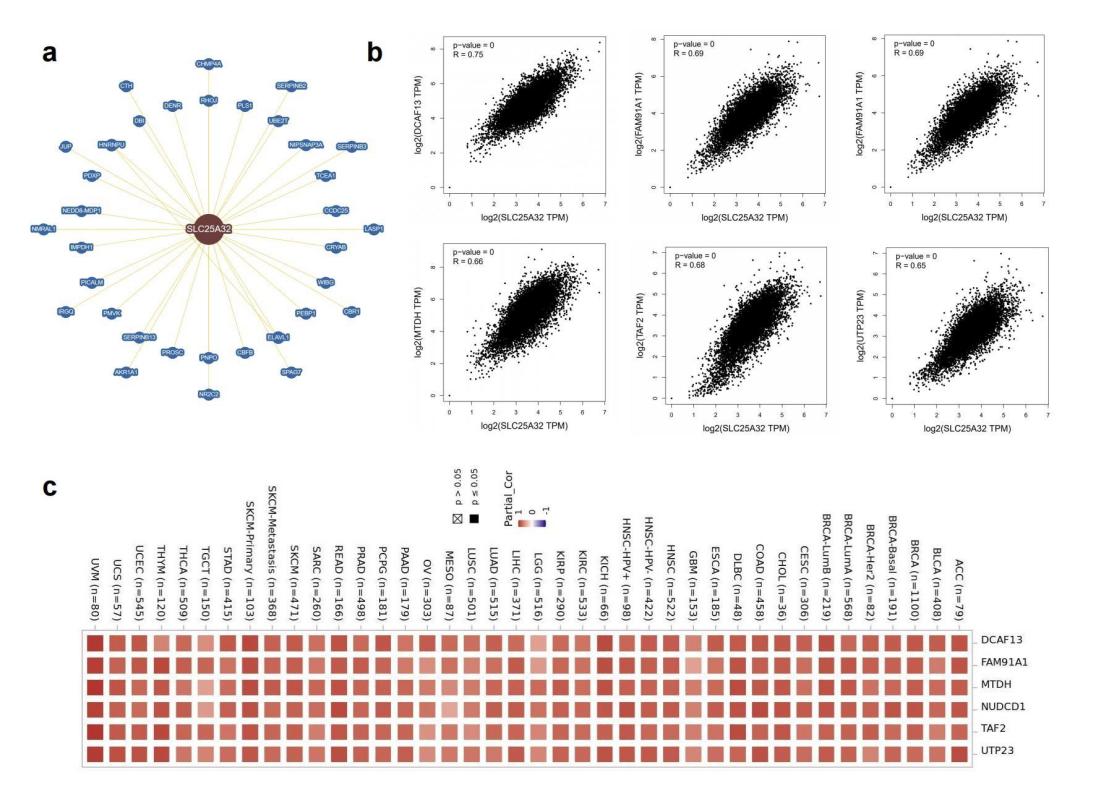


**Supplementary figure S7. The LinkedOmics platform and Metascape were used to further analyse the gene and functional enrichment of SLC25A32 co-expressed in breast cancer**

**
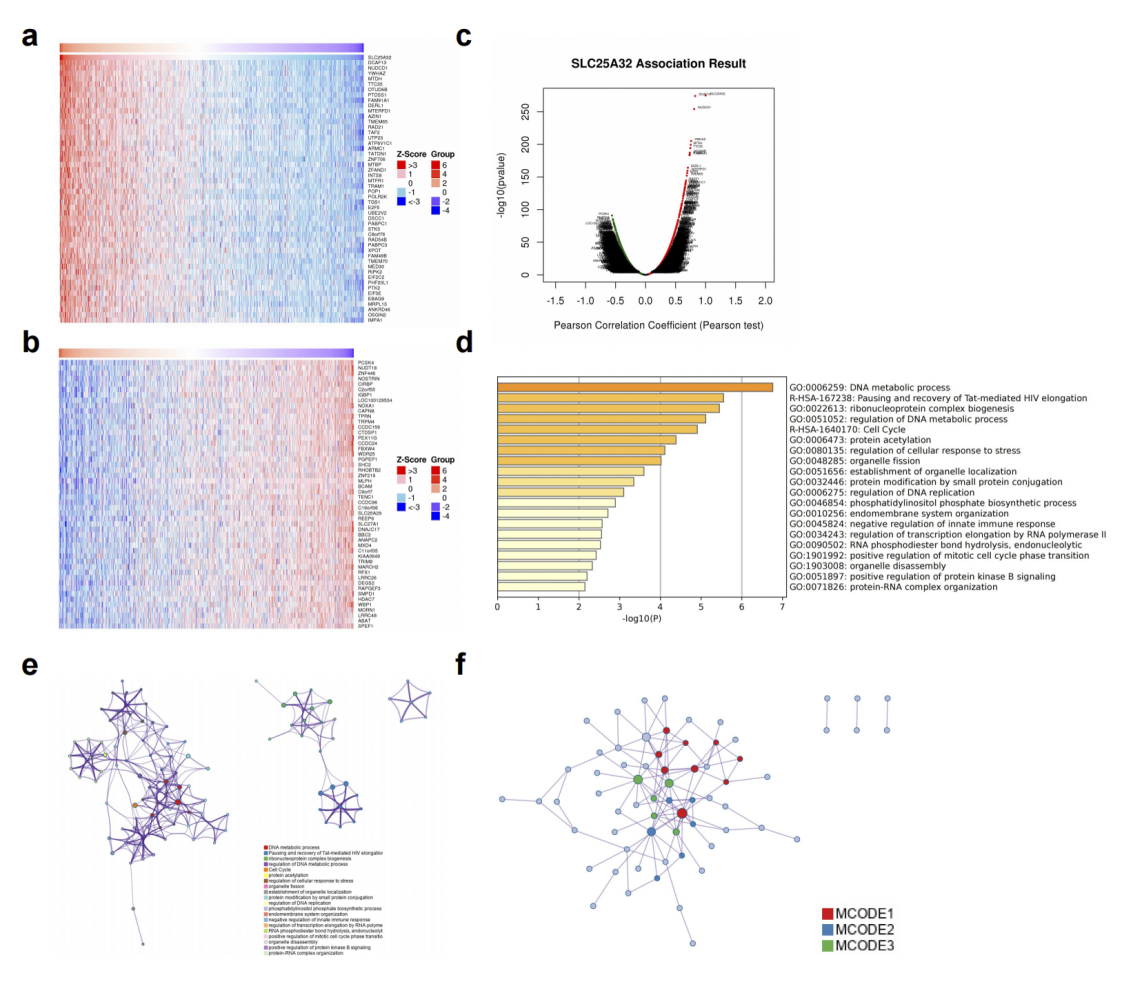
**
